# Transição de cuidados de adolescentes com condições crônicas de saúde e deficiência para os serviços de adultos

**DOI:** 10.1590/0102-311XPT071224

**Published:** 2025-06-09

**Authors:** Dolores Lima da Costa Vidal, Amanda de Carvalho Marques, Martha Cristina Nunes Moreira, Orli Carvalho da Silva, Juliana Marin Fontes, Grace Ferreira de Araujo, Alessandra Augusta Barroso Penna e Costa, Audrey Vidal Pereira

**Affiliations:** 1 Instituto Nacional de Saúde da Mulher, da Criança e do Adolescente Fernandes Figueira, Fundação Oswaldo Cruz, Rio de Janeiro, Brasil.; 2 Universidade Federal Fluminense, Niterói, Brasil.

**Keywords:** Cuidado Transicional, Adolescente, Profissionais de Saúde, Condições de Saúde Crônicas Múltiplas, Pessoas com Deficiência, Transitional Care, Adolescent, Health Personnel, Multiple Chronic Conditions, Persons with Disabilities, Cuidado de Transición, Adolescente, Personal de Salud, Condiciones Crónicas Múltiples, Personas con Discapacidad

## Abstract

Neste artigo, analisamos como os profissionais de saúde dos ambulatórios especializados do Município do Rio de Janeiro, Brasil, que prestam cuidados aos adolescentes com condições crônicas de saúde e deficiência, entendem e realizam a transição de cuidados desse grupo para os serviços de adulto. Participaram, entre o segundo semestre de 2023 e o primeiro trimestre de 2024, 26 profissionais de saúde que atendem adolescentes. Foram utilizados questionários compostos por perguntas abertas e fechadas, através da plataforma Google, e as respostas foram analisadas através da técnica de análise temática. Os resultados apontaram que o conceito de transição de cuidados apresenta empregabilidade imprecisa; que pode ser identificada como transferência, alta, liberação ou desligamento. Dentre os critérios utilizados para caracterizar transição de cuidados, foram levadas em consideração a idade, resolução de queixas e a própria alta. Já as estratégias utilizadas foram: contato direto com o profissional de referência para o serviço de adulto, encaminhamento para a clínica da família visando regulação de vaga, consulta de retorno no serviço de origem, entrega de relatório final, debates entre a equipe multiprofissional e orientações para a família. Em suma, a implementação de um processo de transição de cuidados organizado, planejado e valorizado pelos profissionais da saúde, mostra-se imprescindível para assegurar a defesa do direito à continuidade do cuidado de adolescentes com deficiência e condição crônica para o serviço de adulto, evitando impactos negativos nas condições de saúde dessa população.

## Abstract (en)

This study analyzes how healthcare providers from specialized outpatient clinics in the municipality of Rio de Janeiro, Brazil, who provide care to adolescents with chronic health conditions and disabilities understand and transition care from this group to adult services. From the second half of 2023 to the first quarter of 2024, 26 healthcare providers caring for adolescents participated in this research. Questionnaires with open- and closed-ended questions were used on the Google platform. Their answers were analyzed by thematic analysis. Results showed that the concept of transition of care shows inaccurate employability, which can equate to transfer, discharge, release, or dismissal. The criteria to characterize transition of care considered age, resolution of complaints, and discharge. Strategies included direct contact with the reference professional for the adult service, referral to a family health unit to regulate vacancies, follow-up consultation at the original service, delivery of the final report, debates in the multiprofessional team, and guidance for the family. The implementation of a care transition process organized, planned, and valued by healthcare providers is essential to defend the right to continuous care for adolescents living with disabilities and chronic conditions into the adult service and to avoid negative impacts on their health status.

## Resumen

En este artículo analizamos cómo los profesionales de la salud de las clínicas ambulatorias especializadas de la ciudad de Río de Janeiro, Brasil, que brindan atención a adolescentes con afecciones crónicas de salud y discapacidades entienden y llevan a cabo la transición de la atención de este grupo a los servicios para adultos. Entre el segundo semestre de 2023 y el primer trimestre de 2024, participaron 26 profesionales de la salud que atienden a adolescentes. Se utilizaron cuestionarios compuestos por preguntas abiertas y cerradas en la plataforma Google, y sus respuestas fueron analizadas mediante la técnica de análisis temático. Los resultados mostraron que el concepto de transición de la atención presenta un uso inexacto y puede identificarse como transferencia, alta, liberación o desconexión. Entre los criterios utilizados para caracterizar la transición de la atención, se tuvieron en cuenta la edad, la resolución de las quejas y la propia alta. Se utilizaron las siguientes estrategias: contacto directo con el profesional de referencia para el servicio para adultos, derivación a la clínica de la familia con el objetivo de regular la vacante, cita de regreso al servicio de origen, entrega de informe final, debates entre el equipo multidisciplinario y orientación para la familia. En conclusión, la implementación de un proceso de transición asistencial organizado, planificado y valorado por los profesionales de la salud es fundamental para garantizar la defensa del derecho a la continuidad asistencial de los adolescentes con discapacidad y condiciones crónicas para el servicio de adultos, lo cual evita impactos negativos en las condiciones de salud de esta población.

## Introdução

Transição, semanticamente, significa passagem de um estado de coisas a outro: um momento intermediário entre fases, com demanda de certa extensão temporal. O cuidado à saúde está relacionado à atenção, à responsabilidade e ao desvelo com pessoas ^1^. Quando se justapõem estas palavras, transição e cuidado, torna-se possível entender que o conceito transição de cuidados expressa uma ideia de um processo que demanda atenção, planejamento, intencionalidade, envolvimento e responsabilidades.

Logo, o conceito de transição de cuidados tem sido utilizado para embasar o processo de negociação, organização e pactuação de demandas e necessidades entre os sujeitos envolvidos no cuidado à saúde; tanto durante a passagem da criança ao adolescente, assim como desse para o jovem adulto e ainda do adulto ao idoso.

Neste artigo, a transição de cuidados diz respeito ao processo de passagem do cuidado realizado em instituições de saúde que atendem adolescentes - que podem ser nomeados ora como pessoas com deficiência, ora como adolescentes com condição crônica de saúde - para instituições que ofereçam serviços para jovens adultos ^2^ ^,^ ^3^. A atenção aos adolescentes ou jovens não deve ser confundida com a premissa de que sejam “crianças maiores”. Essa imagem reifica processos de infantilização desse grupo, em uma ótica adultocêntrica, que se encontra com o capacitismo nas interpretações sobre sua existência no mundo ^4^ ^,^ ^5^.

Não utilizamos deficiência e condição crônica como sinônimos. Reconhecemos a deficiência como uma categoria política. No entanto, no campo das práticas profissionais, os adolescentes que são atendidos nos serviços especializados convivendo com condições crônicas de saúde, muitas vezes são identificados como adolescentes com deficiência. Assim, ainda que estes campos não sejam sinônimos, eles possuem pontos de encontro na expressão motora, intelectual, psicossocial e sensorial destes adolescentes. Consideramos que estas nomeações se encontram hoje no vocabulário das instituições de saúde, onde a transição epidemiológica revela o incremento das condições crônicas de saúde, que, segundo Organização Mundial da Saúde (OMS) ^6^, englobam “as deficiências” em seu escopo. 

Além disso, faz total diferença compreender de qual adolescência falamos ao discutir sobre transição de cuidados. Nesse sentido, trata-se de uma adolescência que frequenta, em intensidade, os serviços públicos de saúde desde a infância, por vezes, com necessidades indicadas ainda no pré-natal. São crianças nascidas prematuras ou com diagnósticos que carregam dependências tecnológicas (medicações de alto custo, sondas, fórmulas nutricionais, próteses, órteses, cadeiras de rodas, respiradores), que utilizam inúmeros serviços (terapias de reabilitação, apoio educacional e emocional) incluindo altos custos familiares com o cuidado. 

Importa destacar que a definição da OMS de condição crônica de saúde possibilita reconhecer e não invisibilizar os cuidados contínuos como característicos das necessidades de saúde das crianças prematuras ou com condições de saúde raras, que prosseguem ao longo da vida, se beneficiando dos avanços da engenharia biomédica, atenção vacinal e técnicas cirúrgicas ^6^ ^,^ ^7^. Assim, ao englobar numa definição sanitária “as deficiências” no plural, desconsidera-se a discussão e a definição política da categoria deficiência como um marcador social da diferença, não restrito à saúde, mas pertencente ao campo das lutas políticas por direito a existir na diversidade ^7^. Dessa forma, ressaltamos que não identificamos deficiência como condição de saúde, mas a usamos como uma forma de nomear o público que desafia processos ainda pouco pensados e organizados, de transição de cuidados da adolescência para a vida adulta. 

Nessa perspectiva, pensar e analisar a transição de cuidados da infância para a adolescência e dessa para a vida adulta, a partir do conceito de integralidade do cuidado centrado no sujeito, corrobora para um entendimento de que a deficiência vivenciada por esses adolescentes deve ser considerada, através do modelo social da deficiência e não focado em seu modelo biológico ^4^ ^,^ ^6^ ^,^ ^7^ ^,^ ^8^. Precisamos ainda compreender a experiência com a cronicidade, pois revela a convivência de famílias com uma medicalização da vida, ao buscar respostas sobre a existência de seus filhos, que muitas vezes são anunciados como inviáveis. São situações desafiadoras vividas durante a utilização contínua dos serviços de saúde, podendo influenciar o processo da transição de cuidados desses adolescentes para outros serviços de saúde.

Assim, o entendimento e a reflexão das necessidades individuais e coletivas do sujeito representam a essência da integralidade, compreendendo uma pluralidade de concepções, ações e atitudes que demandam uma análise ampla do sujeito-usuário, considerando suas amplas dimensões, idealizadas e vivenciadas no cotidiano da atenção à saúde ^9^. No que tange ao atendimento integral à saúde de adolescentes com deficiência, Leis como a de *nº 13.146/2015* ^10^ (Estatuto da Pessoa com Deficiência), *nº 8.069/1990* ^11^ (Estatuto da Criança e do Adolescente) e *nº 12.852/2013* ^12^ (Estatuto da Juventude), reforçam, por exemplo, a atenção integral à saúde numa perspectiva ampliada, que visam a construção de políticas públicas, sob a ótica da inclusão de crianças, adolescentes e jovens com deficiência para um acesso universal à saúde através do Sistema Único de Saúde (SUS).

Apesar de os direitos relacionados à saúde desses adolescentes e jovens estarem apropriadamente estabelecidos pelos diferentes marcos regulatórios supracitados, ainda há desafios evidenciando lacunas na prestação de cuidados ^8^. Um deles relaciona-se ao não reconhecimento no território nacional de uma Política de Atenção à Saúde do Adolescente ^13^. E, quando se pensa em adolescentes com deficiência e condição crônica de saúde, onde a tônica do cuidado constitui um componente da sua vida, por vezes desde o nascimento, supomos ser difícil pensar uma mudança do cuidado e de equipe, sem preparo e planejamento.

Nesse sentido, a transição desse cuidado abrange não só o acesso aos serviços de acordo com as demandas de saúde, mas também objetiva que esse cuidado deva ser continuado e considere, em profundidade, os conceitos de adolescências ^14^ e de deficiência. Para isso, uma abordagem ancorada na multidisciplinaridade e conectada com a Rede de Atenção à Saúde (RAS) da pessoa com deficiência se torna fundamental para lidar com as condições de saúde e suas complicações cotidianas. 

Importante atentar que a ausência ou um processo inadequado de transição de cuidados pode trazer consequências para a vida desses jovens adultos, como: interrupção ou abandono de acompanhamento de saúde, inadaptação, lacunas e desencontro de informações; desorganização na oferta de vagas e na gestão da atenção especializada; além de dificuldades do acesso aos direitos sociais, em virtude de dificuldades a documentação médica e social ^2^ ^,^ ^15^.

No Brasil, apesar dessa discussão ser relativamente recente, os trabalhos seguem a tendência dos estudos internacionais ^3^ ^,^ ^16^, nos quais ainda se fragmenta a discussão na transição de cuidados por diagnósticos nosológicos. Logo, o foco se direciona quase exclusivamente pela clínica ou para doença, como por exemplo, anemia falciforme ^17^ e doenças cardíacas congênitas ^18^, e não pelo cuidado em si. Embora a discussão sobre transição de cuidados pensada a partir das doenças tenha sua relevância, defende-se maior pertinência numa reflexão focada na centralidade das adolescências, deficiência e condição crônica de saúde, articulada com a RAS e, consequentemente, com o SUS.

Diante disso, este artigo buscou analisar como os profissionais de saúde dos ambulatórios especializados do Município do Rio de Janeiro, Brasil, que prestam cuidados aos adolescentes com condições crônicas de saúde e deficiência, entendem e realizam a transição de cuidados desse grupo para os serviços de adulto.

## Método

Trata-se de um estudo qualitativo realizado com 26 profissionais de saúde de serviços públicos de saúde do Município do Rio de Janeiro, entre o segundo semestre de 2023 e o primeiro trimestre de 2024. Para responder ao objetivo de compreender as concepções e experiências dos profissionais de saúde sobre a transição de cuidados relacionados aos adolescentes com deficiência e condição crônica de saúde, no Município do Rio de Janeiro, foi organizado um instrumento com perguntas abertas e fechadas que versaram sobre: (1) perfil do profissional; (2); concepção sobre a transição de cuidados; (3) O atendimento de adolescentes; (4) estratégias e alternativas para realização de uma transição de cuidados segura e efetiva de adolescentes para os serviços de adulto. 

Este instrumento auto preenchível foi elaborado em plataforma Google Forms (https://workspace.google.com/intl/pt-BR/products/forms/) e disseminado via WhatsApp, sem utilização de lista de transmissão. Os participantes foram selecionados por conveniência e disponibilidade. Essa disseminação do formulário da pesquisa se deu por meio da rede de universos familiares dos profissionais atuantes e engajados no trabalho em saúde especializado com adolescentes. A equipe de pesquisa foi responsável por essa divulgação, considerando serem eles próprios profissionais especializados na atuação com esses adolescentes. Essa disseminação por rede de universos familiares seguiu uma orientação socioantropológica ^19^. Nessa localização metodológica, assumimos que a propagação da pesquisa e a indicação de participantes acontecem através da intencionalidade das redes de conhecimento, familiaridade com o trabalho, interesse no tema e referência em atuação no atendimento de adolescentes. Há uma aposta alinhada com o campo de significados, proximidade e reciprocidade e, portanto, interesses em construir a pesquisa. Com a intenção de garantir a segurança e confidencialidade dos participantes, eles receberam códigos alfanuméricos visando sua anonimização.

Os critérios de inclusão para o convite a auto-responder o instrumento foram: profissionais de saúde de nível superior; estar atuando, no mínimo, dois anos com adolescentes; e realizar atividades em algum estabelecimento de saúde no município do Rio de Janeiro; sendo critérios de exclusão: licença médica ou férias durante o período de coleta de dados.

Inicialmente as respostas foram codificadas, preservando o sigilo dos participantes. Depois, essas foram tabuladas e organizadas em documento Excel (https://products.office.com/), permitindo leituras seriadas, identificação e agrupamento de informações, sendo de acesso apenas dos pesquisadores. Destacamos que essa organização categórica e com distribuição de frequências descritivas das respostas não caracteriza uma perspectiva quantitativa, mas cumpre a função de organização das informações, localização e categorização das respostas. O foco da pesquisa foi alcançar significados, compreensões e conhecimentos sobre o conceito e práticas de transições de cuidados de adolescentes com deficiência e condições crônicas de saúde.

Utilizou-se a técnica de análise temática ^20^ atendendo etapas como pré-análise, exploração do material, tratamento e interpretação dos resultados, sendo possível identificar padrões e perspectivas acerca do processo de transição de cuidados de adolescentes realizados pelos participantes, que permitiu a elaboração de categorias temáticas, como: (1) a transição de cuidados de adolescentes na perspectiva dos profissionais de saúde: barreiras que emergem; (2) estratégias e alternativas para uma transição de cuidados segura e efetiva de adolescentes para os serviços de adulto.

Assim, após a primeira análise temática desse acervo, foi construído o segundo movimento de interpretação das ausências e presenças de sentidos sobre a transição de cuidados, sem desconsiderar as concepções subjacentes sobre deficiência e adolescências, à luz dos debates teóricos sobre transição de cuidados, deficiência e as tensões entre uma visão especializada e outro encarnado na integralidade de base comunitária. Temos por pressuposto que a atenção à criança tende a embaçar os entendimentos da adolescência como uma fase singular da existência, e quando a adolescência se entrecruza com a presença da deficiência e da condição de saúde crônica, há um incremento de complexidade no campo da atenção. Faz parte dessa complexificação a dificuldade em enxergar esse público para além do setor especializado da saúde. Esse embaçamento se acentua quando se entrecruza com o campo da atenção e práticas de saúde relacionadas às necessidades de saúde referentes à essa transição. 

A pesquisa foi aprovada pelo Comitê de Ética em Pesquisa do Instituto Nacional de Saúde da Mulher, da Criança e do Adolescente Fernandes Figueira, Fundação Oswaldo Cruz (IFF/FIOCRUZ; CAAE 5.989.059) e obteve um total de 60 Termos de Consentimento Livre e Esclarecido (TCLE) respondidos. Entretanto, fez-se necessária a exclusão de 34 termos, pelos seguintes motivos: duplicados (n = 8), sem respostas do questionário (n = 18), e profissionais de saúde de outros municípios (n = 8).

O percurso metodológico foi realizado levando-se em conta tópicos contemplados no guia COREQ (*Consolidated Criteria for Reporting Qualitative Research*) ^21^, versão traduzida para o português, utilizado para relatar dados qualitativos de pesquisa em saúde.

## Resultados e discussão

### Caracterização do perfil dos participantes

Como destacamos na seção anterior, a caracterização do perfil dos 26 profissionais de saúde participantes nos possibilita obter sua localização demográfica e profissional (Tabela 1).

**Tabela 1:** Perfil sociodemográfico dos participantes.

Variáveis	n (%)
Sexo	
Feminino	19 (73)
Masculino	7 (27)
Faixa etária (anos)	
26-35	5 (19)
36-45	13 (50)
46-55	6 (23)
56-65	2 (8)
Cor	
Branca	15 (58)
Parda	10 (38)
Preta	1 (4)
Profissão	
Assistente social	1 (4)
Enfermeiro	4 (15)
Médico(a)	19 (73)
Psicólogo(a)	2 (8)
Formação	
Pós-graduação *lato sensu*/Residência	10 (38)
Mestrado	11 (42)
Doutorado	5 (19)
Tempo de trabalho na instituição (anos)	
1-3	4 (15)
3-5	4 (15)
5-10	2 (8)
10-15	7 (27)
15-20	7 (27)
Acima de 20	2 (8)
Tipo de vínculo empregatício com a instituição	
CLT	2 (8)
Contrato temporário	2 (8)
Servidor público	17 (65)
Outro	5 (19)

CLT: Consolidação das Leis do Trabalho.

Predominou a faixa etária entre 36 e 45 anos, sendo 19 mulheres. Esse predomínio do gênero feminino na força de trabalho na área da saúde é evidenciado ao redor do mundo, tendo em vista a tradicional associação das mulheres com as áreas de cuidado e assistência ^22^ ^,^ ^23^. Quanto ao quesito raça/cor, 15 participantes se autodeclararam brancos (58%), 10 pardos (38%) e 1 preto (4%). Referente à categoria profissional, tivemos 73% da medicina (n = 19); 15% da enfermagem (n = 4), 8% da psicologia (n = 2) e 4% do serviço social (n = 1). O predomínio de profissionais brancos e médicos nos faz refletir sobre a desigualdade, marcada pela questão da raça/cor nas profissões historicamente reconhecidas pela sociedade como de prestígio ^24^.

Quanto ao tempo de vínculo empregatício, 54% dos profissionais (n = 14) possuem entre 10 e 20 anos de experiência na instituição; sendo que 65% são servidores públicos (n = 17). Em relação à formação dos profissionais, 10 (38%) relataram pós-graduação *lato sensu*/Residência, 11 (42%) Mestrado, e 5 (19%), Doutorado. Esse dado deve ser analisado no conjunto com outras características do perfil dos participantes. Destacamos aqui o vínculo mais estável com o serviço público, assim como o tempo desse vínculo. Ou seja, essa maior densidade de práticas e permanência parece remeter a um perfil condizente com profissionais da atenção especializada. 

A transição de cuidados de adolescentes para serviços de adulto: um debate com o acervo da pesquisa

No Brasil, a literatura sobre transição de cuidados é recente e escassa, tratando-se de um conceito ainda impreciso e com significações diversas ^2^ ^,^ ^25^. A terminologia ainda é pouco conhecida e, muitas vezes, confundida com referência e contrarreferência ^24^, ou ainda, com a simples transferência de responsabilidades. 

Os estudos sobre transição de cuidados têm se configurado no eixo internação-domicílio ^26^ ^,^ ^27^ ^,^ ^28^, além de centrados em diagnósticos nosológicos ^17^ ^,^ ^28^ ^,^ ^29^. Portanto, eles são restritos quando relacionados ao acompanhamento ambulatorial entre diferentes níveis de atenção à saúde. As respostas obtidas na pesquisa possibilitaram ratificar tal informação, uma vez que, ao ser compartilhada com os participantes a pergunta sobre o termo utilizado, no serviço, para o desligamento ou finalização do atendimento de adolescentes do ambulatório - alta médica, alta ambulatorial, desligamento, ou finalização do atendimento de adolescentes do ambulatório? - alta médica, alta ambulatorial, desligamento, transferência de cuidados (saída de um serviço de saúde para outro), transição de cuidados (processo contínuo e gradual de preparação para o desligamento de um serviço para outro) e outros -, 20 dos 26 participantes apontaram perspectivas associadas à transferência, alta ou desligamento (Tabela 2). Além disso, quatro dos profissionais participantes (15%) disseram nunca terem ouvido falar no termo transição de cuidados.

**Tabela 2:** Perspectivas teóricas sobre transição de cuidados de adolescentes para os serviços de adulto.

Critérios para o desligamento	n (%)
Transferência de cuidados	11 (42)
Transição de cuidados	5 (19)
Alta ambulatorial	4 (15)
Alta médica	4 (15)
Desligamento	1 (4)

Como os participantes possuem uma longa trajetória em instituições de saúde, possivelmente podemos inferir que não tiveram acesso às informações sobre o conceito de transição de cuidados durante a sua formação, tendo em vista que o respectivo uso é recente no cotidiano das instituições de saúde e de pesquisa no país. No entanto, importa salientar que os conceitos apontados pelos participantes são distintos e possuem implicações diferentes no processo de cuidado, pois remetem a outras concepções. A transferência de cuidados diz respeito à delegação, à cessão, à entrega da responsabilidade, à mudança. Logo, o responsável pelo cuidado deixa de ser um para ser outro; diz respeito à modificação da equipe que presta o cuidado ou até mudança de instituição ^30^.

Na perspectiva teórica, tanto transferência quanto transição de cuidados se apresentam como termos sem consenso. Há autores ^31^ que entendem a transferência de cuidados com base na lógica do encaminhamento, seguido de desresponsabilização pelo usuário. Outros ^32^ ^,^ ^33^ já possuem um entendimento de transferência de cuidados como similar ao da transição, utilizando comumente como termos equiparáveis. O mesmo se observou com os participantes da pesquisa, em que a maioria, 11 (42%), apontou como sendo a forma utilizada para conclusão do acompanhamento do cuidado do adolescente para o serviço de adulto, utilizando transferência como sinônimo de transição de cuidados.

“*Pois a adolescência é fase por natureza vulnerável, de mais difícil identificação com os adultos, uma transição não realizada pode criar muito desamparo e desconfiança*” (P22).

“*Embora no ambulatório em que atuo o termo utilizado seja transferência, acredito que a concepção transição seja válida, pois a equipe busca que o adolescente seja atendido ao menos duas vezes no outro serviço e esteja efetivamente inserido para não ser mais agendado no serviço atual*” (P4).

Com relação aos extratos anteriores, destacamos inicialmente a preocupação com o processo futuro e com a imagem de que se precisa começar a pensar a adolescência ainda na infância, enquanto construção a ser processada pela família e pelos próprios profissionais. Ainda percebemos a imagem da transição de cuidados através de trânsitos processados com continuidade. A evocada “natureza vulnerável da adolescência” pode carregar dificuldades no eixo do vínculo e acolhimento fundamentais na discussão da transição de cuidados. Essas imagens reatualizam estigmas frente à adolescência, como uma barreira ao reconhecimento dessa população ^13^.

Outros participantes apontaram que a transferência está relacionada ao momento da mudança de equipes do cuidado ou da instituição de saúde, no dia da finalização do atendimento. Essa concepção só tem sentido se for articulada a um processo anterior, com planejamento e previsão da data final, que seria o da transferência.

“*Transferência dos cuidados para outro serviço*” (P26).

“*No momento da transferência para o serviço de adulto, transferência para outro serviço pediátrico ou abandono do tratamento*” (P10).

Vejamos que o termo transferência pode resultar em uma concepção de mudança de endereço e localização de cuidado, comprometendo a integralidade e a caracterização de uma rede a ser construída. Tal contexto pode confundir os sentidos a ponto de causar abandonos de tratamento com mudança de locais de referência. Cabe enfatizar que a transição de cuidados prima pelos vínculos e construções de parcerias onde profissionais, famílias e, sobretudo, os adolescentes são mobilizados ^16^.

Com base no conceito de integralidade e considerando a semântica das palavras transição e transferência, assim como a apropriação da terminologia nos artigos pesquisados ^24^ ^,^ ^25^ ^,^ ^26^, defende-se que o conceito de transição de cuidados seja mais adequado quando se refere ao processo que deve ser realizado juntamente com o adolescente, família, equipe e RAS ^34^.

Essa decisão baseia-se na compreensão de que a transição de cuidados não se limita à transferência de uma instituição ou equipe de cuidados para outra. Ela engloba uma série de elementos e intervenções coordenadas que auxiliam adolescentes e suas famílias a transitarem de um modelo de cuidados de adolescentes para o adulto, visando garantir uma transição completa e segura ^33^ ^,^ ^35^ ^,^ ^36^.

Outros conceitos utilizados pelos participantes para se referirem à finalização do acompanhamento de adolescentes, foram os de alta médica, hospitalar e ambulatorial. Trata-se de conceitos tradicionais e históricos ainda muito utilizados, no âmbito da saúde suplementar. Em relação à alta, podem-se identificar concepções como liberação ou desligamento. A liberação refere-se à data de finalização do acompanhamento por um provedor, profissional ou instituição de saúde. Já o desligamento é considerado um termo mais amplo relativo ao processo de liberação do cuidado de uma instituição de saúde, seja após a alta ou transferência para outra instituição ^37^ ^,^ ^38^.

A diversidade dos termos empregados para definir a mudança de fase do cuidado do adolescente para um serviço de adulto aponta para a necessidade do estabelecimento de etapas e diretrizes coletivas que precisam ser discutidas e planejadas com os sujeitos envolvidos no processo de transição de cuidados do adolescente com as RAS ^34^.

Contudo, ainda que o termo transição de cuidados não esteja generalizado enquanto uma concepção de acompanhamento para a continuidade do cuidado para os serviços de adulto, verificou-se um esforço por parte dos participantes de estabelecerem maneiras de analisar ou teorizar esse processo; o que leva a inferir que possuem certo consenso sobre a importância de realização da transição de cuidados.

Dentre o grupo investigado, 17 (65%) participantes não se consideram especialistas em adolescentes, embora apontando que realizam acompanhamento e cuidado de adolescentes.

“*Não há uma equipe dedicada exclusivamente à assistência deste público*” (P5).

“*Neste ambulatório são atendidas todas as faixas etárias. Não existe atendimento específico ao adolescente*” (P20).

As respostas citadas nos remetem aos sentidos desse trânsito entre infância e adolescência. Ao que parece, faz-se urgente compreender que crianças não são adolescentes, e apesar de óbvio este destaque, o mesmo não se configura nas práticas de saúde. Predomina uma equivalência ou continuidade entre as idades, o que faz embaçar as necessidades distintas que tais períodos da vida exigem. A linguagem, a perspectiva e o entendimento sobre necessidades e abordagens distintas entre estes sujeitos do cuidado, se complexifica frente à presença da deficiência e da condição crônica de saúde. Mecanismos de infantilização comprometem a construção do protagonismo dos adolescentes, e quando reunidos às estruturas adultocêntricas e capacitistas ^4^ ^,^ ^5^ podem produzir exclusões sucessivas. 

Um efeito mais flagrante dessas exclusões acontece por meio da ausência de uma identificação por parte dos participantes, que não se reconhecem como profissionais especialistas no atendimento de adolescentes. E não defendemos neste contexto a necessidade de uma equipe para atenção “exclusiva” aos adolescentes, porém ressaltamos que as demandas da adolescência não são as mesmas da infância - ainda que o campo de atuação pediátrica compreenda toda infância e adolescência.

Uma interpretação necessária diz respeito à necessidade de considerar a diversidade que configura o adolescente com deficiência e condição crônica de saúde. Assim, a ausência de atenção direcionada a esses adolescentes durante o processo de transição de cuidados − possivelmente devido à escassez de políticas públicas e serviços qualificados e voltados para suas necessidades − pode ainda contribuir para o aumento das disparidades e da dificuldade de acesso aos serviços, comprometendo a efetividade do princípio da integralidade e longitudinalidade do cuidado.

### Estratégias e alternativas para uma transição de cuidados segura e efetiva de adolescentes para os serviços de adulto

Os critérios apontados pelos participantes para a transição de cuidados dos adolescentes para os serviços adultos (Tabela 3), como a faixa etária e resolução do problema, não apenas desempenham um papel crucial na organização desse processo, como também possibilitam uma identificação das necessidades do usuário ao longo dessa transição. Definir critérios e organizar o processo de transição permite aos profissionais de saúde personalizar os cuidados, garantindo continuidade de ações planejadas e consistentes ao longo dos diferentes estágios do desenvolvimento dos adolescentes; além de evitar tensões, riscos e prejuízos para a sua saúde.

**Tabela 3:** Critérios e dificuldades para a realização da transição de cuidados segundo os participantes.

Critérios para o desligamento	n (%)
Idade	16 (62)
Resolução do problema	7 (27)
Não há critérios definidos	1 (4)
Formas complementares	25 (96)
Obstáculos na migração ou no acolhimento nos serviços de adulto	12 (46)
Escassez de vagas ou à demora no acesso	9 (35)

Em contrapartida, os critérios para efetivar a transição de cuidados acabam sendo mais fruto de ações individuais que coletivas. Percebeu-se uma ausência de estratégias coletivas ou estabelecidas de maneira institucional que facilitassem ou direcionassem a transição de cuidados de um serviço pediátrico para o segmento de adulto. Através da análise realizada, identificou-se que os participantes possuem diversas maneiras de realizar a transição de cuidados. Destaca-se ainda que apenas um dos profissionais (4%) afirmou não estabelecer critérios para realizar a transição de cuidados. Quando o adolescente completa 18 anos, ele informa “*sem preparo, como uma notícia dada nos últimos momentos*” (P8).

Dentre os 26 participantes, 96% indicaram formas de transicionar o cuidado, que acabam sendo complementares entre si.

“*Ofereço encaminhamento e relatório de acompanhamento, para que haja tempo hábil para conseguir novo atendimento e o mesmo* [adolescente] *não ficar sem assistência*” (P18).

“*Durante o atendimento a equipe médica anuncia que em breve o adolescente será encaminhado para outro serviço em virtude da idade*” (P4).

Ao analisar os critérios apontados pelos profissionais, notou-se que 16 (62%) consideraram a idade como principal fator para transição de cuidados dos adolescentes. Entretanto, é importante sinalizar que a transição para o atendimento adulto necessita considerar não apenas a idade de forma isolada. Para além dessa, importa pensar sobre a aquisição de habilidades e competências individuais do adolescente, a prontidão e autonomia junto de suas famílias para o processo do início e continuidade da transição de cuidados ^35^ ^,^ ^36^.

As mudanças no cenário epidemiológico dos últimos anos no Brasil podem ser ratificadas pelos dados do Instituto Brasileiro de Geografia e Estatísticas (IBGE) ^39^, que apresentou uma estimativa aproximada da existência de 18,6 milhões de pessoas com idade igual ou superior a 2 anos, com deficiência em pelo menos uma de suas funções. Na prática da atenção, tem-se observado que essas crianças e adolescentes estão vivendo cada vez mais. Dessa forma, são necessárias análises prospectivas que garantam políticas públicas para essa população com objetivo de proporcionar acesso adequado e com qualidade à saúde. Se antes pensar processos de transição para a vida adulta era algo pontual e esporádico, atualmente é algo necessário e urgente para a realidade brasileira.

Vale destacar que 7 (27%) participantes sinalizaram que a transição de cuidados acontece com a resolução do problema. Uma imagem ainda muito pautada na “cura” ou resolução de queixas pontuais pode não acompanhar o cenário de transição epidemiológica, onde a presença da deficiência e da condição de saúde crônica exige uma análise que considere o viver com a cronicidade como mediadora da vida. Essas crianças que passaram a viver com a cronicidade, tornar-se-ão adolescentes e adultos que, ao acompanhar a própria saúde, terão que manejar o viver com a cronicidade ^40^; onde reconhecer um corpo com outras funcionalidades prescinde mediar processos capazes de atender as necessidades complexas que envolvam diagnósticos de condições crônicas e raras de saúde e temporalidade estendida pela convivência ^41^. Assim, a categoria cronicidade engloba, mas não se reduz ou identifica à condição de saúde, nem seus tempos de curso e manifestação, agudo ou crônico. Ela evoca temporalidade na experiência corporal, nos encontros, com o que é interpretado e negociado intersubjetivamente. A cronicidade significa “viver com e gerenciar” a vida e o cuidado perene, e desigualmente distribuído, como expressão e característica mediadora do viver a vida e seus custos. “Cronicidade e deficiência” são categorias com fronteiras em fricção permanente, para quem vive com uma condição de saúde rara e complexa ^41^.

Os profissionais que já enfrentam essa realidade com os adolescentes com deficiência e condição crônica conseguem entender a necessidade desse processo, de tal forma que todos valorizaram a transição de cuidados, ainda que não vivenciem atividades programadas e reconhecidas institucionalmente como tal. Percebemos intencionalidade e preocupação com a continuidade do cuidado por parte deles, quer seja pela perspectiva de segurança da atenção e do cuidado, quer seja pela possibilidade de buscar a garantia da autonomia do adolescente perante seu cuidado. 

“*…é importante dar continuidade ao seguimento do paciente e direcionar para as novas demandas tanto da adolescência quanto da vida adulta*” (P2).

“*...especialmente por conta da autonomia e autorresponsabilidade pela saúde, que em muitos casos de atendimento do adolescente pelo pediatra, ainda é parcialmente delegado aos pais*” (P15).

Além disso, os profissionais sinalizaram que a ausência de determinadas ações, como o registro e dados sobre o perfil dos adolescentes, dificulta processos que refletem tanto no serviço de origem quanto no serviço de adulto. Isto é, repercute na falta de planejamento na oferta de serviços, o que, por sua vez, também dificulta a entrada de novos usuários nos serviços especializados.

Conforme apontado, 12 (46%) participantes identificaram a saída do serviço como a principal dificuldade para os usuários, devido aos obstáculos no acolhimento e no processo de migração para os serviços de adulto. Por outro lado, nove (35%) apontaram a escassez de vagas ou a demora no acesso como um desafio para a entrada dos usuários nos serviços especializados que atendem adolescentes e jovens adultos. Importa ressaltar que essas dificuldades relacionadas aos serviços de adulto interferem nos critérios utilizados pelos profissionais dos ambulatórios especializados quando realizam o processo de transição de cuidados ^42^.

Dessa maneira, este estudo não só apresenta as concepções dos profissionais de saúde sobre o conceito de transição de cuidados, mas também registra que estabelecer estratégias para transicionar de modo seguro os cuidados de adolescentes com deficiência ou com condições crônicas de saúde para o serviço de adulto, previne impactos negativos na saúde deles e qualifica os processos no âmbito do SUS.

No entanto, esta análise apresenta limitações relacionadas ao reduzido número de participantes que finalizaram o preenchimento do questionário e à restrição à realidade do Município do Rio de Janeiro, o que impede generalizações. Dessa forma, é interessante ampliar e aprofundar conhecimentos sobre transição de cuidados a partir de estudos futuros.

## Considerações finais 

Ainda que os profissionais reconheçam a importância da transição de cuidados de adolescentes com deficiência e condições crônicas de saúde, ganham destaque algumas inquietações. Mesmo que não tenha sido objeto deste estudo, vale refletir que a ausência de estratégias coletivas e organizadas por parte dos serviços pediátricos, para o estabelecimento de parâmetros e recomendações para a realização do processo de transição de cuidados uniformizado no SUS, pode caracterizar apontamentos úteis para investigações futuras.

Em relação às concepções teóricas, os participantes entendem a transição de cuidados a partir do uso de conceitos como transferência, alta, liberação e desligamento. Alguns profissionais expressam ausência de critérios e estratégias para esse processo. Entretanto, dentre os critérios utilizados por estes profissionais para caracterizar ações de transição de cuidados dos adolescentes, foram pontuadas variáveis como idade, resolução do problema/queixa e a própria alta. E dentre as estratégias, pode-se observar ações centradas em condutas individuais; articulação e contato direto com o profissional de referência para os serviços de adulto; encaminhamento para clínica da família visando regulação da marcação de consultas; retorno para avaliação no serviço pediátrico; entrega de relatório final atrelado à idade de 18 anos; debates caso a caso entre a equipe pediátrica; e orientações para a família.

Em suma, a defesa do direito de adolescentes com ou sem deficiência em relação à continuidade do cuidado no serviço de adulto precisa ser assegurada, antes do término do seu atendimento no serviço pediátrico. Há um reconhecimento pelos profissionais de que a ausência do processo de transição de cuidados em ambulatórios especializados pode trazer consequências negativas, como a descontinuidade do cuidado desses jovens com deficiência e condição crônica de saúde.

Desse modo, a implementação de um processo de transição de cuidados organizado e planejado de modo prévio e contínuo, além de interferir diretamente na RAS, cuja materialização torna-se explícita a partir da disponibilização da oferta de vagas para o acesso dessa população aos serviços de adultos, afetando diretamente na abertura de novas vagas nos serviços de adolescentes na atenção especializada, mostra-se imprescindível para evitar impactos negativos nas condições de saúde destes adolescentes com deficiência e suas respectivas famílias.

## References

[1] Pinheiro R, Mattos RA (2005). Cuidado: as fronteiras da integralidade..

[2] Canadian Association of Pediatric Health Centres (2016). A guideline for transition from paediatric to adult health care for youth with special health care needs: a national approach..

[3] Kanhere S, Joshi SM (2023). Transition of care in epilepsy.. Indian J Pediatr.

[4] Dias FS, Moreira MCN, Silva LN (2023). Deficiência e capacitismo: uma agenda nacional inconclusa para a 17ª Conferência Nacional de Saúde.. Cad Saúde Pública.

[5] Moreira MCN, Dias FS, Mello AG, York SW (2022). Gramáticas do capacitismo: diálogos nas dobras entre deficiência, gênero, infância e adolescência.. Ciênc Saúde Colet.

[6] Organização Mundial da Saúde (2003). Cuidados inovadores para condições crônicas: componentes estruturais de ação. Relatório mundial..

[7] Moreira MCN, Albernaz LV, Sá MRC, Correia RF, Tanabe RF (2017). Recomendações para uma linha de cuidados para crianças e adolescentes com condições crônicas complexas de saúde.. Cad Saúde Pública.

[8] Ambrosio RBA, Pedroso GC, Puccini RF, Souza FIS (2023). Crianças e adolescentes com deficiência: desafios na formação médica.. Rev Bras Educ Méd.

[9] Oliveira IC, Cutolo LRA (2018). Integralidade: algumas reflexões.. Rev Bras Educ Méd.

[10] Brasil (2015). Lei nº 13.146, de 6 de julho de 2015. Institui a Lei Brasileira de Inclusão da Pessoa com Deficiência (Estatuto da Pessoa com Deficiência).. Diário Oficial da União.

[11] Brasil (1990). Lei nº 8.069, de 13 de julho de 1990. Dispõe sobre o Estatuto da Criança e do Adolescente e dá outras providências.. Diário Oficial da União.

[12] Brasil (2013). Lei nº 12.852, de 5 de agosto de 2013. Institui o Estatuto da Juventude e dispõe sobre os direitos dos jovens, os princípios e diretrizes das políticas públicas de juventude e o Sistema Nacional de Juventude - SINAJUVE.. Diário Oficial da União.

[13] Lopez SB, Moreira MCN (2013). Quando uma proposição não se converte em política?: o caso da Política Nacional de Atenção Integral à Saúde de Adolescentes e Jovens - PNAISAJ.. Ciênc Saúde Colet.

[14] Moraes BR, Weinmann AO (2020). Notas sobre a história da adolescência.. Estilos Clín.

[15] Brabo TSAM, Silva MEF, Maciel TS (2020). Gênero, sexualidades e educação: cenário das políticas educacionais sobre os direitos sexuais e reprodutivos de jovens e adolescentes.. Práxis Educativa.

[16] Wenger JK, Niemann M (2020). Continue the conversation: a complex care pediatrician&apos;s perspective on improving healthcare transitions for pediatric neurology patients.. Semin Pediatr Neurol.

[17] Peres J (2016). Análise do preparo para a transição de adolescentes que vivem com doença falciforme para a vida adulta: um estudo de método misto.

[18] McLoughlin A, Matthews C, Hickey TM (2018). &raquo;,» &reg;,® &sect;,§ &shy;,­ &sup1;,¹ &sup2;,² &sup3;,³ &szlig;,ß &THORN;,Þ &thorn;,þ &times;,× &Uacute;,Ú &uacute;,ú &Ucirc;,Û &ucirc;,û &Ugrave;,Ù &ugrave;,ù &uml;,¨ &Uuml;,Ü &uuml;,ü &Yacute;,Ý &yacute;,ý &yen;,¥ &yuml;,ÿ &para;,¶ They&apos;re kept in a bubble &raquo;,» &reg;,® &sect;,§ &shy;,­ &sup1;,¹ &sup2;,² &sup3;,³ &szlig;,ß &THORN;,Þ &thorn;,þ &times;,× &Uacute;,Ú &uacute;,ú &Ucirc;,Û &ucirc;,û &Ugrave;,Ù &ugrave;,ù &uml;,¨ &Uuml;,Ü &uuml;,ü &Yacute;,Ý &yacute;,ý &yen;,¥ &yuml;,ÿ &para;,¶ : healthcare professionals&apos; views on transitioning young adults with congenital heart disease from paediatric to adult care.. Child Care Health Dev.

[19] Velho G, Nunes E (1978). A aventura sociológica..

[20] Bardin L (2016). Análise de conteúdo..

[21] Souza VR, Marziale MH, Silva GT, Nascimento PL (2021). Tradução e validação para a língua portuguesa e avaliação do guia COREQ.. Acta Paul Enferm.

[22] Pereira AV, Tardem GCRR, Vidal DLC, Alves VH, Vieira BDG, Cortez EA (2023). Profile of healthcare workers during the COVID-19 pandemic in Rio de Janeiro: a web survey study.. Online Braz J Nurs.

[23] World Health Organization (2019). Delivered by women, led by men: a gender and equity analysis of the global health and social workforce..

[24] Valério ACO, Bezerra WC, Santos VS, Leite JD, Farias MN, Santos SMB (2021). Racismo e participação social na universidade: experiências de estudantes negras em cursos de saúde.. Cadernos Brasileiros de Terapia Ocupacional.

[25] Acosta AM (2016). Transição do cuidado de pacientes com doenças crônicas: do serviço de emergência para o domicílio.

[26] Bernardino E, Sousa SM, Nascimento JD, Lacerda MR, Torres DG, Gonçalves LS (2022). Cuidados de transição: análise do conceito na gestão da alta hospitalar.. Esc Anna Nery Rev Enferm.

[27] Gomes VC, Lanzoni GMM, Cechinel-Peiter C, Santos JLG, Melo ALSF, Magalhães ALP (2023). Quality of child and adolescent care transitions considering the presence of chronic disease.. Rev Bras Enferm.

[28] Acosta AM, Lima MADS, Pinto IC, Weber LAF (2020). Transição do cuidado de pacientes com doenças crônicas na alta da emergência para o domicílio.. Rev Gaúcha Enferm.

[29] Mackie AS, Fournier A, Swan L, Marelli AJ, Kovacs AH (2019). Transition and transfer from pediatric to adult congenital heart disease care in canada: call for strategic implementation.. Can J Cardiol.

[30] Praisner T, Santos CL (2021). A transferência de cuidados: Um dispositivo para análise do cuidado compartilhado na rede de Atenção Psicossocial.. Cadernos Brasileiros de Saúde Mental.

[31] Alves M, Melo CL (2019). Transferência de cuidado na perspectiva de profissionais de enfermagem de um pronto-socorro.. REME - Revista Mineira de Enfermagem.

[32] Castro CMCSP, Marques MCMP, Vaz CROT (2022). Comunicação na transição de cuidados de enfermagem em um serviço de emergência de Portugal.. Cogitare Enferm.

[33] Zucchetti M, Severo IM, Echer IC, Borba DSM, Nectoux CLS, Azzolin KO (2022). Validation of manual to complement the transition of care at discharge from intensive care.. Rev Gaúcha Enferm.

[34] Ministério da Saúde (2014). Implantação das Redes de Atenção à Saúde e outras estratégias da SAS..

[35] Pastura PSVC, Paiva CG (2018). Transição dos cuidados de pacientes com doenças crônicas da pediatria para a medicina de adultos: práticas de um hospital terciário no Brasil.. Revista de Pediatria SOPERJ.

[36] Belga SMMF, Jorge AO, Silva KL (2022). Continuidade do cuidado a partir do hospital: interdisciplinaridade e dispositivos para integralidade na rede de atenção à saúde.. Saúde Debate.

[37] Khalaf Z (2023). Improving patient handover: a narrative review.. Afr J Paediatr Surg.

[38] Agência Nacional de Saúde Suplementar (2020). Consórcio de indicadores de qualidade hospitalar..

[39] Gomes I (2023). Pessoas com deficiência têm menor acesso à educação, ao trabalho e à renda.. Agência IBGE Notícias.

[40] Moreira MEL, Goldani MZ (2010). A criança é o pai do homem: novos desafios para a área de saúde da criança.. Ciênc Saúde Colet.

[41] Moreira MCN (2022). Configurações do ativismo da parentalidade atípica na deficiência e cronicidade.. Ciênc Saúde Colet.

[42] Shamambo L, Niemann M, Jonas R, Douglass LM (2020). Mentorship for youth with chronic neurological conditions in the digital era: an innovative approach to supporting transition.. Semin Pediatr Neurol.

